# Cellular and Humoral Immune Responses after Immunisation with Low Virulent African Swine Fever Virus in the Large White Inbred Babraham Line and Outbred Domestic Pigs

**DOI:** 10.3390/v14071487

**Published:** 2022-07-07

**Authors:** Lynnette C. Goatley, Rachel H. Nash, Catherine Andrews, Zoe Hargreaves, Priscilla Tng, Ana Luisa Reis, Simon P. Graham, Christopher L. Netherton

**Affiliations:** The Pirbright Institute, Ash Road, Pirbright, Woking GU24 0NF, UK; lynnette.goatley@pirbright.ac.uk (L.C.G.); r_h_d@hotmail.co.uk (R.H.N.); cmaa133@aol.co.uk (C.A.); zoehargreaves@hotmail.co.uk (Z.H.); priscilla.tng@pirbright.ac.uk (P.T.); ana.reis@pirbright.ac.uk (A.L.R.); simon.graham@pirbright.ac.uk (S.P.G.)

**Keywords:** african swine fever virus, immune response, antibody, cell-mediated immunity, T-cell response, inbred pigs

## Abstract

African swine fever virus is currently present in all of the world’s continents apart from Antarctica, and efforts to control the disease are hampered by the lack of a commercially available vaccine. The Babraham large white pig is a highly inbred line that could represent a powerful tool to improve our understanding of the protective immune responses to this complex pathogen; however, previous studies indicated differential vaccine responses after the African swine fever virus challenge of inbred minipigs with different swine leukocyte antigen haplotypes. Lymphocyte numbers and African swine fever virus-specific antibody and T-cell responses were measured in inbred and outbred animals after inoculation with a low virulent African swine fever virus isolate and subsequent challenge with a related virulent virus. Surprisingly, diminished immune responses were observed in the Babraham pigs when compared to the outbred animals, and the inbred pigs were not protected after challenge. Recovery of Babraham pigs after challenge weakly correlated with antibody responses, whereas protective responses in outbred animals more closely correlated with the T-cell response. The Babraham pig may, therefore, represent a useful model for studying the role of antibodies in protection against the African swine fever virus.

## 1. Introduction

African swine fever virus (ASFV) is a large double-stranded DNA virus that causes a lethal haemorrhagic disease in domestic pigs and wild boar. Case fatality rates approach 100% after infection with virulent isolates; however, infection with attenuated viruses obtained from the field or generated by targeted gene deletion can show a much less severe disease course, and in many cases, recovered animals are fully protected from subsequent challenge with related virulent viruses [1,2,3]. Twenty-four ASFV genotypes (I through XXIV) have been identified based on sequencing of 400 bp of the 3′ end of the *B646L* gene within the 170 to 190 kbp viral genome [4]. In addition, at least eight serogroups have been characterised through a series of cross-protection experiments, the results of which correlate with the sequences of the *EP402R* and *EP153R* genes [5,6,7]. However, ASFV genotypes are poor predictors of cross-protection [8,9,10], and serogroups do not explain the protection afforded by live attenuated viruses (LAV) that lack the genetic loci responsible [3,8,10,11,12].

Infection of pigs with attenuated viruses induce both cellular and humoral immune responses [13], and evidence exists for the importance of both arms of the adaptive response for protection [14,15,16,17,18]. LAV-immunised pigs typically have circulating T-cells capable of secreting cytokines [19], proliferating in response to recall antigen [20] and ASFV-specific cytolytic activity [21,22]. Antibodies capable of neutralising a virus [23], lysing infected cells via antibody-dependent cellular cytotoxicity (ADCC) [24] or complement-dependent cytotoxicity [25] and inhibiting the haemadsorption of erythrocytes to infected macrophages [5] have been described. Nevertheless, the relative importance of these effector responses in protection is unclear, and matters are complicated by the different combinations of viruses used for immunisation and challenge. Depletion of CD8α cells from pigs immunised with the low virulent, non-haemabsorbing genotype I OUR T88/3 isolate abrogates protection afforded against the highly virulent genotype I OUR T88/1 isolate [18]. In addition, the secretion of IFNγ in response to the recall antigen correlated to protection mediated by OUR T88/3 against virulent genotype I and genotype X viruses [8]. In contrast, the protection afforded by the E75-CV1 haemabsorbing genotype I virus against homologous and heterologous challenges correlated with the proliferation of CD8 cells, not IFNγ secretion [9]. Experiments with a non-haemabsorbing genotype I BA71ΔCD2v virus, generated by targeted gene deletion, strongly suggested a role for the cellular immune response in heterologous protection [10,12]. However, the ASFV-specific antibody response more closely correlated to protection than the ASFV-specific cellular immune response in pigs inoculated with the genotype XX gene deleted Pretoriuskop/96/4Δ9GL virus [26]. In passive transfer experiments, ADCC activity correlated positively with clinical outcomes against heterologous challenge (genotype I serum, genotype X challenge), although the authors were unable to measure other ASFV-specific antibody effector functions [16]. Passive transfer of serum from E75-CV1-immunised and E75-challenged pigs protected naïve animals from severe disease [17], and colostrum from sows or serum from pigs recovered from the genotype I Dominican Republic 1979 isolate of ASFV protected neonatal pigs from homologous challenge [15].

Taken together, these data suggest that both cellular and humoral responses play a role in the protective response against ASFV, but teasing apart the relative importance of different effector mechanisms is difficult due in part to the variety of different model systems employed to study them. This relatively poor understanding of the immune responses required for protective immunity restricts the development of both LAV and subunit vaccines against ASF. The Babraham Large White pig is a highly inbred line of approximately 85% homozygosity [27] that are sufficiently inbred to permit adoptive transfer experiments [28,29,30]. Swine leukocyte antigen class I (SLA-I) tetramers for SLA-1*14:02 and SLA-2*11:04 have been developed and used to quantify the magnitude and kinetics of the T-cell response to influenza in Babraham pigs [31,32]. We have recently characterised the breadth of the T-cell response to ASFV infection in Babraham pigs [33], and therefore, this inbred line could be used for a detailed analysis of the protective immune response to ASF. However, previous experiments with NIH minipigs suggested that the SLA haplotype may play a role in protection as NIH *dd* minipigs were fully protected against OUR T88/1 challenge after OUR T88/3 inoculation, whereas the NIH *cc* line was not [18]. An immunisation and challenge experiment was, therefore, carried out to test the OUR T88/3–OUR T88/1 model in the Babraham line and examine lymphocyte dynamics and immune responses after immunisation and challenge in detail.

## 2. Materials and Methods

### 2.1. Animal Studies

Animal experiments were carried out under the Home Office Animals (Scientific Procedures) Act (1986) (ASPA) and were approved by the Animal Welfare and Ethical Review Board (AWERB) of The Pirbright Institute. The animals were housed in accordance with the Code of Practice for the Housing and Care of Animals Bred, Supplied or Used for Scientific Purposes, and bedding and species-specific enrichment were provided throughout the study to ensure high standards of welfare. Through careful monitoring, pigs that reached the scientific or humane endpoints of the studies were euthanised by an overdose of anaesthetic. All procedures were conducted by Personal License holders who were trained and competent and under the auspices of Project License PPL70/8852.

Seven female (animals 896, 905, 906, 907, 910, 914 and 917) and ten male (897, 899, 900, 908, 909, 911, 912, 913, 915 and 916) fifteen-week-old Babraham pigs were bred at Animal Plant Health Agency, APHA Weybridge, UK. Twelve pigs were randomly assigned to a group that were immunised with low virulent OUR T88/3, and the remaining five were inoculated with phosphate buffer saline. Eight-week-old outbred female Landrace × Large white × Hampshire pigs were obtained from a high health farm in the UK. Lameness in pig AV78 was treated with 0.04 mL/kg penicillin/streptomycin and 0.02 mL/kg meloxicam (Metacam) for three days, followed by a single injection of 7.5 mg/kg enrofloxacin (Baytril). Scoring of clinical signs and macroscopic lesions assessed at post-mortem were as described previously [8,34].

### 2.2. Cells and Viruses

Tissue-culture adapted Ba71v [35], low virulent OUR T88/3, virulent OUR T88/1 [36] and virulent Georgia 2007/1 [37] ASFV strains were cultured and titrated using end point dilution on bone-marrow-derived macrophages as described previously [33]. Challenge doses for animal experiments were confirmed by back titration. Virus in the blood and tissues was titrated using quantitative PCR [38]. Peripheral blood mononuclear cells (PBMC) were prepared from heparinised blood using Histopaque and cultured in RPMI, GlutaMAX, HEPES supplemented with 10% foetal calf serum, 1 mM sodium pyruvate, 50 µM 2-mercaptoethanol, 100 IU/mL penicillin and 100 µg/mL streptomycin (RPMI/10).

### 2.3. ELISA

Fixed cell ELISA on Ba71v infected Vero cells and sandwich ELISA against IFNα were carried out as previously described [33,39]. A competitive ELISA against ASFV p30 was obtained from Innovative Diagnostics (Grabels, France) (ASFC-5P) and porcine IL-10 sandwich ELISA from Thermo Scientific (KSC0101). Levels of IFNα, TNFα, IFNγ, IL-1β, IL-10, IL-12p40, IL-4, IL-6 and IL-8 were analysed using Cytokine and Chemokine 9-Plex Porcine ProcartaPlex™ Panel 1 (Thermo Fisher Scientific, Waltham, MA, USA) on a Bio-Plex 200 System (BioRad Watford, UK).

### 2.4. ELISpot

Interferon gamma (IFNγ) ELIspot was carried out as described [33]. Briefly, peripheral blood mononuclear cells were incubated overnight on multiwell plates coated with capture antibody in the presence of virus, mock inoculum, PHA or media alone. The following day spots were visualised using biotinylated anti-IFNγ antibody and streptavidin alkaline phosphatase. Spots were counted using an S6 Immunospot Analyser (Cellular Technology Limited (Shaker Heights, OH, USA) and converted to the number of spot forming cells per million cells.

### 2.5. Flow Cytometry

#### 2.5.1. Whole Blood Labelling

Whole blood labelling was carried out using a two-step wash protocol in 15 mL Falcon tubes. Primary antibodies (Supplementary Tables S1 and S2, Supplementary Figures S1 and S2) diluted in flow buffer (Ca/Mg free PBS with 2% heat-inactivated foetal calf serum) were added in a 50 µL volume to 100 µL of blood, incubated for 15 min at room temperature and then washed twice with flow buffer. Secondary antibodies diluted in flow buffer were added in a 50 µL volume for 15 min at room temperature. In total, 2 mL of RBC Lysis/Fixation Solution (BioLegend, San Diego, CA, USA) was added to each sample for 30 min at room temperature in the dark. Finally, cells were washed twice more with flow buffer before resuspending in a final volume of 500 μL before running on a MACSQuant Analyser 10 (Miltenyi Biotec, Bergisch Gladbach, Germany) and analysing the data (Supplementary Figures S1 and S2) using FlowJo 10 (Becton, Dickinson and Company, Franklin Lakes, NJ, USA).

#### 2.5.2. Intracellular Cytokine Staining

One million PBMC in RPMI/10 at 5 × 10^6^ cells/mL were incubated overnight in 96 U-bottomed plate wells with media alone, 5 × 10^5^ HAD of the indicated virus (OUR T88/1 or Georgia 2007/1) or an equivalent volume of mock inoculum. Fresh media supplemented with brefeldin A (5 μg/mL, BioLegend) was then added to cells the following morning, along with phorbol 12-myristate 13-acetate (100 ng/mL, (Merck Life Science UK Limited, Gillingham, Dorset, UK) and ionomycin (2 µg/mL, Merck) to the positive controls. After a further 4 h incubation, cells were stained for flow cytometry using panels shown in Supplementary Tables S3 to S5. All staining was carried out in a 50 µL final volume of flow buffer supplemented with 0.2% sodium azide (Supplementary Tables S3–S5). In total, 100,000 live lymphocytes were collected using an LSR Fortessa (Becton, Dickinson and Company), and data were analysed using FlowJo 10 (Supplementary Figures S3–S5).

#### 2.5.3. Proliferation Assays

PBMC were washed twice with PBS and then resuspended in PBS containing 5 μM Cell-Trace Violet (Thermo Fisher) and incubated for 20 min at 37 °C. Dye was quenched with ten volumes of warm RPMI/10, cells then pelleted by centrifugation and one million cells plated out in 100 µL with and without antigen, as described above. Cells were stained for flow cytometry five days later as above, using the panel shown in Supplementary Table S6. Flow cytometry data were analysed using FlowJo 10 (Supplementary Figure S6).

### 2.6. Statistics

Statistical analysis was carried out using Prism GraphPad 9. Unless stated otherwise, a repeated measures (RM) mixed effect model with Sidak’s multiple comparisons test was used to test for statistical differences within and between groups of Babraham animals immunised with PBS (n = 5) or OUR T88/3 (n = 12). An RM mixed effect model with Sidak’s multiple comparison test was also used to test for statistical differences within and between Babraham pigs immunised with PBS (n = 5), those that were immunised with OUR T88/3 and recovered after challenge (n = 5) and those that did not recover (n = 7). Data points between 0 and 23 days post immunisation with OUR T88/3 were used for these analyses when all groups contained at least four data points. Analysis of CD21+ cell numbers within the groups of animals that were fully protected was undertaken using one-way ANOVA.

## 3. Results

### 3.1. Large-White Inbred Babraham Pigs Are Not Protected by Immunisation with OUR T88/3

**Figure 1:**
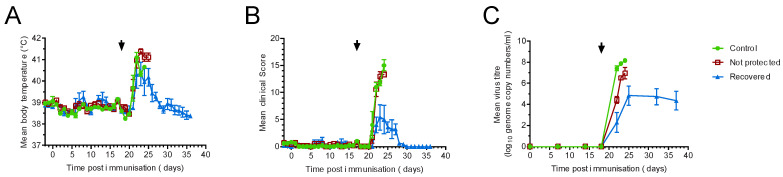
Clinical and virological parameters of Babraham pigs. Inbred Babraham animals were inoculated with low virulent ASFV isolate OUR T88/3 (red and blue) or PBS control (green) and then challenged with highly virulent OUR T88/1 eighteen days later (arrows). Five animals that recovered after challenge are indicated in blue and the seven that did not are in red. Body temperatures (**A**) and clinical signs (**B**) were scored daily and blood samples taken for virus titration on the indicated days (**C**). Viraemia was determined by quantitative PCR. Error bars represent the standard error from the mean.

Twelve inbred Babraham pigs were immunised with 10,000 TCID_50_ of ASFV OURT88/3 by the intramuscular route, and five control animals were immunised with a sham inoculum. Pig 912 developed swelling on one joint 14 days after immunisation, but this did not lead to lameness or develop into a necrotic lesion. After 18 days, all pigs were challenged intramuscularly with 10,000 HAD_50_ of ASFV OURT88/1. Following challenge, all animals showed non-specific clinical signs such as raised body temperature (Figure 1A, Figure S7A–C), inappetence and lethargy (Figure 1B, Figure S7D–F). All of the controls and seven of the immunised animals reached their humane endpoint between five and seven days after challenge (22–24 days post-immunisation), while the remaining five immunised animals recovered and were clinically normal at the end of the experiment, 17 days post-challenge. Four days post-challenge, the animals that ultimately recovered had slightly lower clinical scores than both the controls (*p* = 0.0139) and those that did not recover (*p* = 0.0296; RM two-way ANOVA). Non-specific pathological signs and macroscopic lesions commonly seen in animals suffering acute ASF were observed in pigs at post-mortem that reached humane endpoints, including hydropericardium, ascites, hyperemic splenomegaly and lymphadenopathy. Haemorrhagic lymphadenopathy was observed in the gastro-hepatic and renal lymph nodes in some animals. No differences were observed between the controls and the immunised animals that were euthanised between four and six days post-challenge. All of the animals were viraemic after challenge with OUR T88/1 (Figure 1C, Figure S7G–I). Control animals showed higher viral load in the bloodstream than immunised animals regardless of clinical outcome, and recovered pigs did not clear the virus and were still viraemic at the end of the experiment. There was no correlation between the clinical outcomes and the sex of the animals (*p* = 0.3444 Log-rank (Mantel–Cox) test)).

**Figure 2:**
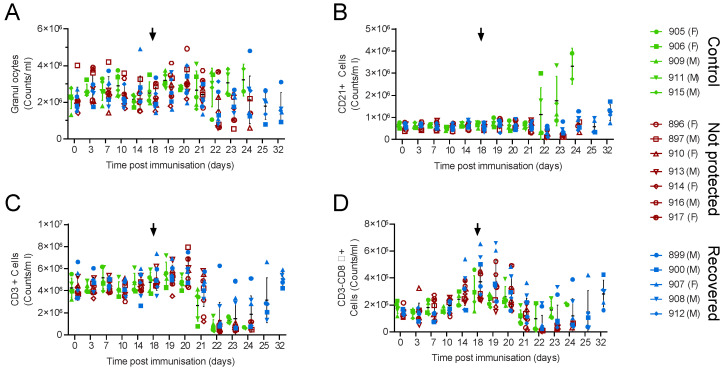
Blood cell numbers in Babraham pigs. Inbred Babraham animals were inoculated with low virulent ASFV isolate OUR T88/3 (red and blue) or PBS control (green) and then challenged with highly virulent OUR T88/1 eighteen days later (arrows). Blood samples were taken on the indicated day, labelled with antibodies and the number of granulocytes (**A**), CD21+ (**B**), CD3+ (**C**) and CD3-CD8α+ cells (**D**) determined by volumetric flow cytometry. Animals immunised with OUR T88/3 that recovered after challenge are indicated as blue symbols and those that did not as dark red symbols. Lines indicate the mean, and error bars indicate the standard deviation from that mean.

To understand the effect of immunisation and challenge on T-cell dynamics, numbers of different blood cell types were determined by flow cytometry (Figure 2). Antibody against CD3 was used to identify T-cells and the combination of antibodies against CD4, CD8α, CD25 and the γδ T-cell receptor (TCR) were used to identify γδ T-cells (CD3+γδTCR+), naïve CD4 cells (CD3+γδTCR-CD4+CD8α-), antigen-experienced CD4 cells (CD3+γδTCR-CD4+CD8α+), activated and regulatory CD4 T-cells (CD3+γδTCR-CD4+CD25+). Antibody against CD8α also allowed the discrimination of populations that include CD8 cytotoxic lymphocytes (CD3+γδTCR-CD8α+CD4-) and NK cells (CD3-CD8α+). Anti-CD21 was used separately to assess changes in conventional B-cells [40]. Granulocytes, T-cells and CD21+ cells in the circulation did not change in Babraham pigs after immunisation with OUR T88/3 or PBS control. Although there appeared to be a trend towards elevated numbers of CD3-CD8α+ cells 14 and 18 days post immunisation, no differences were observed between control and immunised animals at any time point, suggesting this was due to natural variation. After challenge, the numbers of circulating granulocytes did not change significantly; however, numbers of most other cell types began to decrease three and four days post-challenge (21 to 22 days post immunisation), concomitant with the appearance of clinical signs. Numbers of CD21+ cells appeared to increase in the control group relative to the numbers at challenge; however, due to the variation in the data, this was not significantly different from the immunised pigs (RM two-way ANOVA). Lower numbers of circulating cells generally correlated with clinical signs; for example, pigs 906 and 911 (control) and 896 and 914 (immunised) were the first to show clinical signs and had lower numbers of T-cells and CD3-CD8α+ cells. Likewise, 899 had relatively few clinical signs and maintained numbers of T-cell numbers throughout. Cell numbers recovered to similar levels to those found prior to challenge in the animals that recovered.

**Figure 3:**
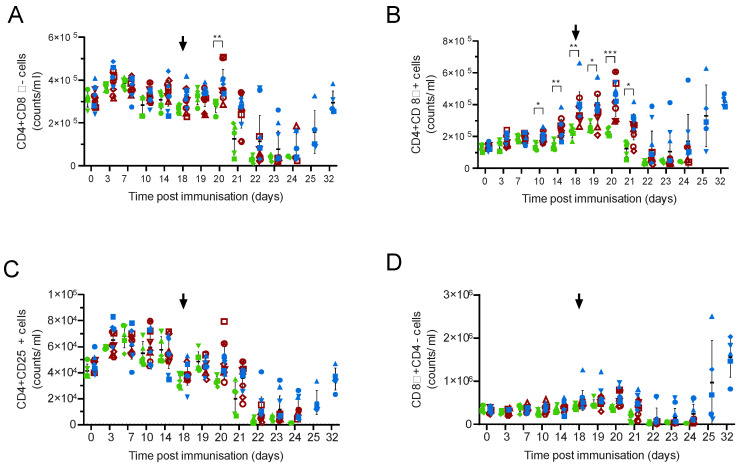
T-cell numbers in Babraham pigs. Inbred Babraham animals were inoculated with low virulent ASFV isolate OUR T88/3 (red and blue) or PBS control (green) and then challenged with highly virulent OUR T88/1 eighteen days later (arrows). Blood samples were taken on the indicated day, labelled with antibodies and the number of CD3+γδTCR-CD4+CD8α- (**A**), CD3+γδTCR-CD4+CD8α+ (**B**), CD3+γδTCR-CD4+CD25+ (**C**) and CD3+γδTCR-CD8α+CD4- (**D**) determined by volumetric flow cytometry. Animals immunised with OUR T88/3 that recovered after challenge are indicated as blue symbols and those that did not as dark red symbols, and individual animals are labelled as shown in the legend of Figure 2. Lines indicate the mean, and error bars indicate the standard deviation from that mean. * *p* < 0.05, ** *p* < 0.01, *** *p* < 0.001. Individual pigs are labelled as in Figure 2.

Individual CD3+ T-cell subsets followed similar patterns to total CD3+ cells (Figure 3; Figure S8), although elevated numbers of both activated and memory T-cells (CD4+CD8α+) and populations that include cytotoxic lymphocytes (CD8α+ CD4-) were observed two weeks post-challenge (Figure 3B,D). Increased numbers of CD4+CD8α+ cells were observed in the immunised animals when compared to the controls between 10 and 20 days post immunisation (M-EA, *p* = 0.0259 to 0.0002); however, differences were not seen between the animals that recovered and those that did not when they were analysed as separate groups. No trends in numbers of CD4+CD25+ cells, which include regulatory T-cells (Figure 3C), or γδ TCR+ or γδTCR+CD8α (Figure S2A,B) were observed other than the general decrease after challenge. The percentage of CD8α+ γδTCR+ cells increased as a proportion of the total number of γδT-cells in some animals after challenge (Figure S2C), but this was not significant and is likely linked to the overall lymphopenia. Further attempts to subdivide cell populations with CD25 did not reveal any trends that related to the clinical outcomes (not shown).

**Figure 4:**
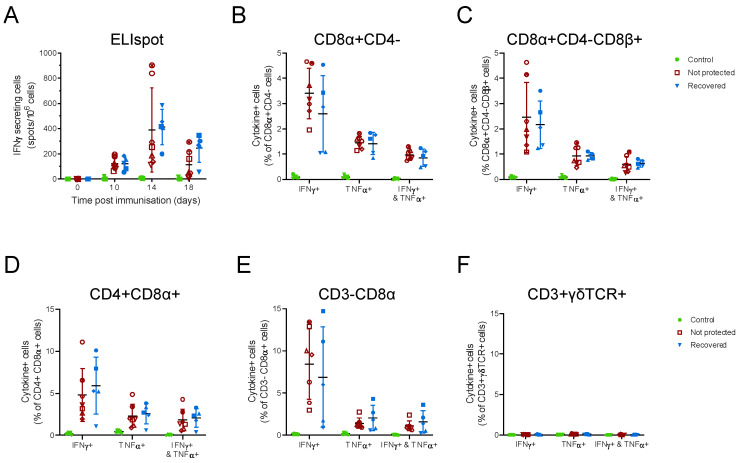
Cellular responses to ASFV in Babraham pigs. Inbred Babraham animals were inoculated with low virulent ASFV isolate OUR T88/3 (red and blue) or PBS control (green) and then challenged with highly virulent OUR T88/1 eighteen days later. Blood samples were taken on the indicated day (**A**) or prior to challenge, 18 days post immunisation (**B**–**F**). IFNγ secreting cells were enumerated by ELISpot (**A**) or after labelling with antibodies for analysis by flow cytometry to identify CD8α+CD4- (**B**), CD8α+CD4-CD8β+ (**C**), CD4+CD8α+ (**D**), CD3-CD8α+ (**E**), or CD3+γδTCR+ cells (**F**). The proportion of each cell type expressing IFNγ, TNFα or both cytokines was determined using ICS (**B**–**D**). Animals immunised with OUR T88/3 that recovered after challenge are indicated as solid blue symbols and those that did not as open dark red symbols and control as green. Lines indicate the mean, and error bars indicate the standard deviation from that mean.

**Figure 5:**
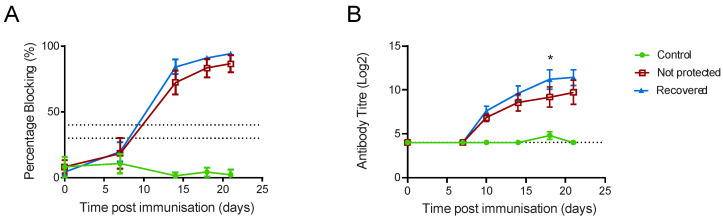
Antibody responses to ASFV in Babraham pigs. Inbred Babraham animals were immunised with low virulent ASFV isolate OUR T88/3 (red and blue) or PBS control (green) and then challenged with highly virulent OUR T88/1 eighteen days later. Blood samples were taken on the indicated day and serum analysed with a competitive ELISA against CP204L/p30 (**A**) or by immunoperoxidase assay on infected Vero cells (**B**). Lines indicate the mean, and error bars indicate the standard deviation from that mean. The upper and lower dashed lines on Panel A represent the manufacturer’s positive and negative cut-offs, while the dashed line on Panel B indicates the limit of detection, * *p* < 0.05.

ASFV-specific cellular immune responses were detected 10 days post immunisation by IFNγ ELIspot (Figure 4A) and the phenotype of responding cell types determined by flow cytometry prior to challenge. The main secretors of IFNγ and TNFα were CD8α+CD4- T-cells (Figure 4B), CD8αCD4+ T-cells (Figure 4D) and CD3-CD8α+ cells (Figure 4E), and no responses were seen from γδ T-cells (Figure 4F). No responses were seen to ASFV in control animals, and no differences were observed in any cell types between animals that survived challenge with virulent OUR T88/1 and those that did not. Attempts to further subdivide T-cells based on expression of CD8β did not reveal any patterns related to clinical outcome (Figure 4C). This showed that, unlike in outbred pigs [8], secretion of IFNγ in response to recall antigen did not predict protection in inbred Babraham pigs immunised with OUR T88/3. ASFV-specific antibody responses determined with a competitive ELISA against the highly immunogenic CP204L/p30 protein (Figure 5A) suggested slightly higher antibody responses in immunised pigs that recovered after challenge, and this was confirmed by fixed cell ELISA (Figure 5B).

Experiments with OUR T88/3 deletion mutants suggested a link between survival after challenge and levels of IL-10 in the serum [41]; however, very little IL-10 was detected before challenge or three days later (Figure S9). Two of the control animals (906 and 911) had IFNα in the serum, and these were the two animals that had the highest viraemia at that time (7.9 log_10_ genome copies/mL). Luminex analysis did not reveal any other cytokines in the serum (not shown), and this suggested that cytokine levels in the circulation did not explain or correlate with the difference in the observed clinical outcomes.

### 3.2. OUR T88/3 Induces High Levels of Protection against Genotype I, but Not Genotype II, Challenge in Outbred Pigs

**Figure 6:**
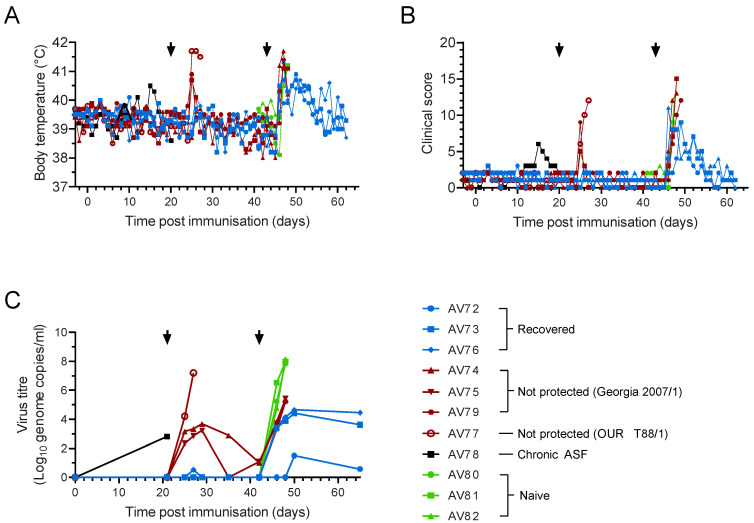
Clinical and virological parameters in outbred pigs. Outbred animals were inoculated with low virulent ASFV isolate OUR T88/3 (red, blue or black) and then challenged with virulent OUR T88/1 (red and blue) 21 days later. All surviving animals, as well as three naïve controls (green), were then challenged with highly virulent Georgia 2007/1 21 days after OUR T88/1 challenge. Body temperature (**A**) and clinical signs (**B**) were scored daily and blood samples taken for virus titration on the indicated days (**C**). Viremia was determined by quantitative PCR. Arrows indicate the challenge with OUR T88/1 and Georgia 2007/1.

The failure of OUR T88/3 to induce protection in Babraham pigs was unexpected, and therefore, to confirm these findings, the immunisation and challenge protocol was repeated in outbred pigs. A group of eight animals were immunised with 10,000 TCID_50_ of ASFV OURT88/3 by the intramuscular route. One pig (AV78) had slightly elevated temperatures compared to the others (Figure 6A) and thirteen days after immunisation had developed swelling on the joint of the front left leg and a swollen popliteal lymph node on the rear left leg (Figure 6B). AV78 then became lame on the rear leg, which did not respond to treatment and, therefore, was euthanised 20 days post immunisation. Necropsy confirmed hyperplasia of the popliteal and inguinal lymph nodes, but no other macroscopic lesions and subsequent analysis demonstrated the animal was viraemic (Figure 6C). It is, therefore, likely that AV78 suffered from chronic ASF.

The remaining seven pigs were then challenged with 10,000 HAD_50_ of ASFV OURT88/1 twenty-one days after immunisation with OUR T88/3. Three animals (AV74, AV75 and AV77) showed non-specific clinical signs such as high temperature, lethargy and inappetence three days post-infection; however, for AV74 and AV75, this lasted a single day. However, humane endpoints for AV77 were reached six days post-challenge, and the animal was euthanised. Viraemia in AV74 and AV75 reached a peak of 10^3.7^ and 10^3.2^ genome copies/mL eight days post-challenge, whereas AV77 had viraemia of 10^7.2^ genome copies/mL at termination.

To test heterologous protection between genotype I and genotype II the surviving six pigs, along with three naïve animals, were then challenged with 1000 HAD_50_ virulent Georgia 2007/1 twenty-one days after challenge with OUR T88/1. All of the animals began to develop clinical signs between four and seven days post-infection. All the naïve animals, as well as AV74, AV75 and AV79, reached their humane end point six and seven days post-challenge and had macroscopic lesions typical for acute ASF. AV72 showed four days at 40 °C or above but only one day of high fever and inappetence. AV73 and AV76 had body temperatures close to or above 40 °C for up to a week but only showed one or two days when they were disinterested in food. All three animals were clinically normal when the experiment was ended three weeks after the Georgia 2007/1 challenge. AV73 and AV76 survived Georgia 2007/1 challenge but with prolonged clinical signs showing significant lung haemorrhage and congestion; AV76 also suffered moderate hyperemic splenomegaly.

All of the animals challenged with Georgia 2007/1 became viraemic, with the naïve pigs reaching 10^7.8^ to 10^8.1^ genome copies/mL. Animals that were not protected showed lower viraemia (10^5.4^ to 10^5.2^) than the controls six days post-challenge, and the maximum viraemia in the animals that survived challenge with Georgia 2007/1 challenge was 10^4.7^. AV72, which showed only transitory clinical signs after Georgia challenge, had a maximum titre of 10^1.5^, showing a rough correlation between viraemia and clinical outcome. The degree of protection against OUR T88/1 did not predict subsequent protection against Georgia 2007/1 as AV74 and AV75 showed transient clinical signs and were viraemic after challenge with OUR T88/1, but AV79 did not.

**Figure 7:**
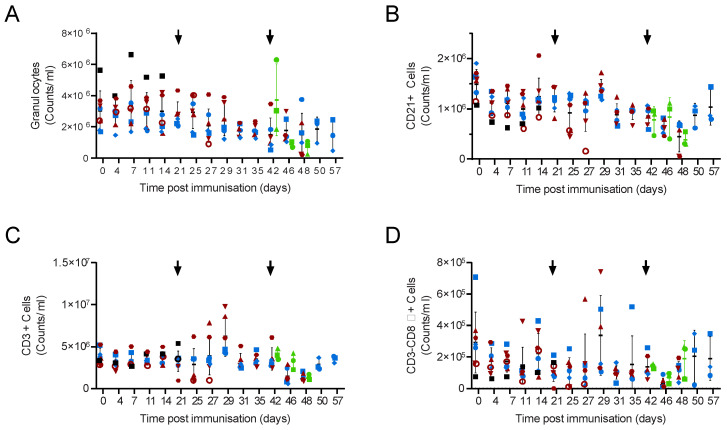
Blood cell numbers in outbred pigs. Animals were inoculated with low virulent ASFV isolate OUR T88/3 (red, blue and black) and then challenged twenty-one days later with highly virulent OUR T88/1, followed by Georgia 2007/1 twenty-one days after that (arrows). Blood samples were taken on the indicated day and the number of granulocytes (**A**), CD21+ (**B**), CD3+ (**C**) and CD3-CD8α+ cells (**D**) determined by volumetric flow cytometry. Animals immunised with OUR T88/3 that recovered after challenge with Georgia 2007/1 are indicated with solid blue symbols and those that did not with solid dark red symbols. Animal AV77 that was not protected against OUR T88/1 is shown as a red open circle and AV78 that suffered chronic ASF and was not challenged with OUR T88/1 as a solid black square. Naïve animals challenged with Georgia 2007/1 are indicated in green. Lines indicate the mean, and error bars indicate the standard deviation from that mean.

**Figure 8:**
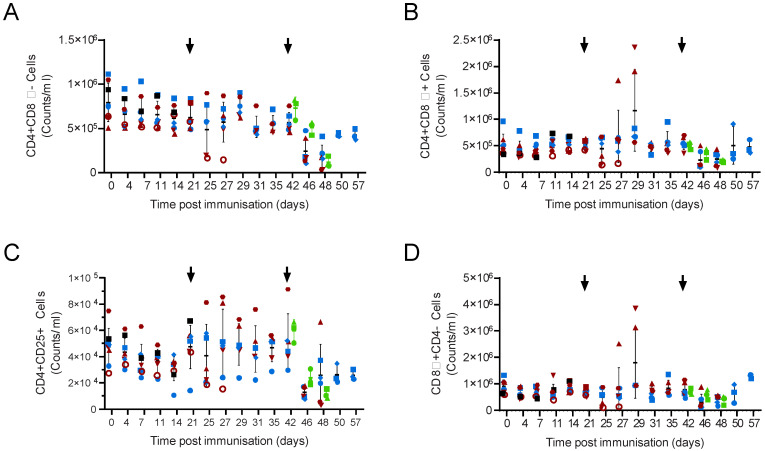
Blood cell numbers in outbred pigs. Pigs, inoculated with low virulent ASFV isolate OUR T88/3 (red and blue) and then challenged twenty-one days later with highly virulent OUR T88/1, followed by Georgia 2007/1 twenty-one days after that (arrows). Blood samples were taken on the indicated day, labelled with antibodies and the number of CD4+CD8α- (**A**), CD4+CD8α+ (**B**), CD4+CD25+ (**C**) and CD8α+CD4- (**D**) cells determined by volumetric flow cytometry. Animals immunised with OUR T88/3 that recovered after challenge with Georgia 2007/1 are indicated with solid blue symbols and those that did not with solid dark red symbols. Animal AV77 that was not protected against OUR T88/1 is shown as a red open circle and AV78 that suffered chronic ASF and was not challenged with OUR T88/1 as a solid black square. Naïve animals challenged with Georgia 2007/1 are indicated in green. Lines indicate the mean, and error bars indicate the standard deviation from that mean.

Numbers of circulating granulocytes, CD21+, CD3+ and CD3-CD8α cells (Figure 7) followed similar patterns to those seen in the Babraham animals. Granulocytes were relatively unaffected after immunisation and the subsequent challenges, and AV78, which suffered chronic ASF, had a higher numbers of granulocytes than the other animals both before and after immunisation. Numbers of other cell types decreased in animals showing clinical signs after challenge and were elevated in those that were recovering and were still viraemic from ASF. There was no increase in circulating CD21+ cells in naïve outbred animals after challenge with Georgia 2007/1, which differed from that seen in the control Babraham animals infected with OUR T88/1. Increases in circulating CD3 cells in animals AV74 and AV75 (Figure 7C) after challenge could be accounted for by the elevated numbers of both CD4+CD8α+ (Figure 8B) and CD8α+CD4- (Figure 8D) cells. Unfortunately, whole blood labelling for γδTCR failed, and therefore, it is possible that an increase in these cells also contributed to the increase in total CD3+ cells, but this did not occur in Babraham pigs recovering from OUR T88/1. Numbers of CD8α+CD4- cells, but not CD4+CD8α+ cells, were elevated in the three surviving animals 15 days post-challenge with Georgia 2007/1.

**Figure 9:**
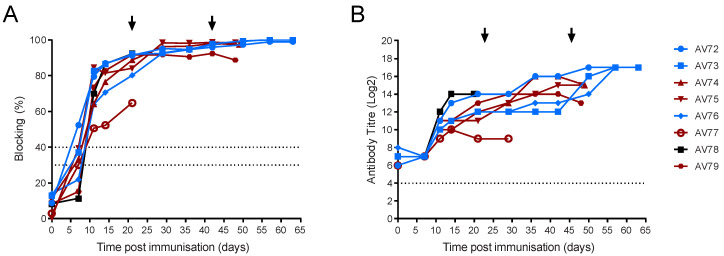
Antibody responses to ASFV in outbred pigs. Pigs were inoculated with low virulent ASFV isolate OUR T88/3 (red, blue and black) and then challenged twenty-one days later with highly virulent OUR T88/1, followed by Georgia 2007/1 twenty-one days after that (arrows). Blood samples were taken on the indicated day and serum analysed with a competitive ELISA against CP204L/p30 (**A**) or by immunoperoxidase assay on infected Vero cells (**B**). Animals that recovered after challenge with Georgia 2007/1 are indicated in blue and those that did not in dark red. AV77 that did not recover after OUR T88/1 challenge is indicated as an open red circle and AV78 that suffered chronic ASF as a black square. The upper and lower dashed lines on Panel A represent the manufacturer’s positive and negative cut-offs, while the dashed line on Panel B indicates the limit of detection for the fixed cell ELISA.

**Figure 10:**
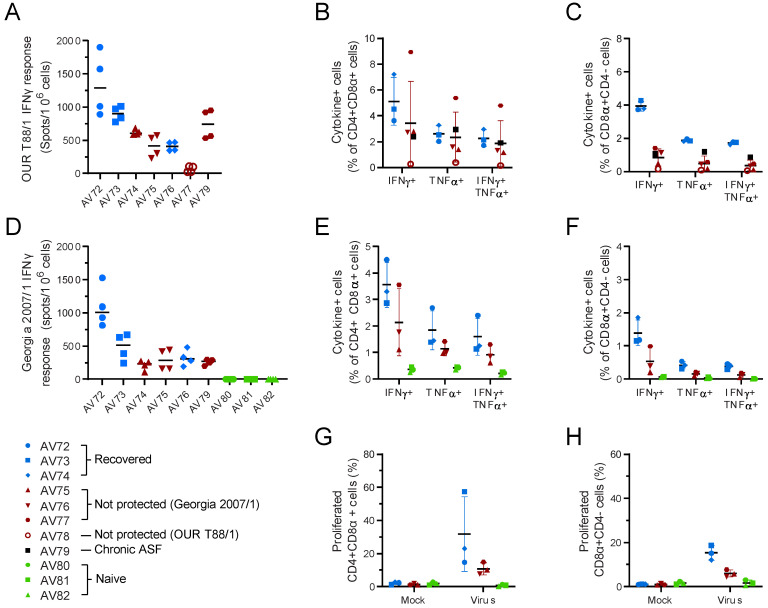
Cellular responses to ASFV in outbred pigs. Animals were inoculated with low virulent ASFV isolate OUR T88/3 and then challenged twenty-one days later with highly virulent OUR T88/1, followed by Georgia 2007/1 twenty-one days after that. Blood samples were taken prior to challenge with OUR T88/1 on day 21 (**A**–**C**) or Georgia 2007/1 on day 42 (**D**–**H**). IFNγ secreting cells were enumerated by ELISpot (**A**,**D**) or flow cytometry (**B**,**C**,**E**,**F**) after stimulation with OUR T88/1 (**A**–**C**) or Georgia 2007/1 (**D**–**F**). The proportion of CD4+CD8α+ (**B**,**E**) or CD8α + CD4- cells expressing IFNγ, TNFα or both cytokines was determined using ICS. PBMCs purified before Georgia 2007/1 challenge were stimulated for 6 days with Georgia 2007/1 or a mock inoculum and the proportion of CD4+CD8α+ (**G**) and CD8α+CD4- (**H**) cells proliferating after 6 days stimulation with Georgia 2007/1 identified. Animals that recovered after challenge with Georgia 2007/1 are indicated in blue and those that did not in dark red. AV77 that did not recover after OUR T88/1 challenge is indicated as an open red circle and AV78 that suffered chronic ASF as a black square. Lines indicate the mean, and the error bars indicate the standard deviation from that mean.

ELISA showed that all of the immunised animals developed antibody responses to p30/CP204L, but the percentage blocking in animal AV77 did not reach the levels seen in the other animals (Figure 9A). This was confirmed by titration on fixed infected cells as ASFV-specific antibody titres in AV77 were at least four-fold lower than in the other pigs (Figure 9B). There was an increase in ASFV-specific antibody titres in most of the animals that survived both the OUR T88/1 and Georgia 2007/1 challenge (Figure 9B). As well as poor antibody responses, AV77 also failed to mount a robust cellular immune response with low numbers of ASFV-specific IFNγ secreting cells compared to the other animals (Figure 10A); this was reflected in lower ASFV-specific CD4+CD8α+ and CD8αCD4- cells (Figure 10B,C). However, there was no clear difference in the measured immune responses of animals AV74 and AV75, which were viraemic and showed clinical signs after OUR T88/1 challenge, when compared to the other four animals that did not. Pre-OUR T88/1 challenge antibody titres (*p* = 0.0151, Welch’s *t*-test) and ASFV-specific IFNγ secreting cells (*p* < 0.0001, Welch’s *t*-test) were lower in the Babraham pigs that were immunised with OUR T88/3 compared to the outbred animals. Challenge with OUR T88/1 did not lead to a significant increase in antibody titres or circulating ASFV-specific IFN-γ secreting cells (not shown).

No differences were detected in the measured antibody responses prior to Georgia challenge between the animals that survived and those that did not (Figure 9). However, differences were observed between the cellular immune responses, with the animal showing the best clinical picture (AV72) having higher total numbers of ASFV-specific IFNγ secreting cells prior to challenge than the other animals (Figure 10D). Although analysis of the ELIspot data did not reveal differences between the animals that survived and those that did not when analysed as a group, differences were observed in individual T-cell subsets. Higher percentages of ASFV-specific CD4+CD8α+ and CD8αCD4- cells (Figure 10E,F), as well as more robust proliferation (Figure 10G,H), were observed in animals that recovered after Georgia challenge when compared to those that did not. Further analysis of T-cell subsets based on labelling for CD25, CD62L and CCR7 to identify effector T-cells did not reveal additional patterns relating to the clinical outcome with respect to Georgia challenge (Figure S10). A slightly higher proportion of CD4+CD8α+ cells were also CD62L+ in animals AV74 and AV75 that were still viraemic at challenge; however, this was not mirrored in the expression of CCR7. The majority of ASFV-specific CD4+CD8α+ T-cells did not express either CD62L or CCR7 consistent with classification as effector T-cells.

## 4. Discussion

Inbred animals are valuable tools for studying infectious diseases and have the potential to reduce the number of animals required to obtain a given scientific objective, a key part of the 3Rs. However, it is important to ascertain whether the inbred line is representative of the wider outbred population and in the context of ASFV, we have demonstrated that the Babraham pigs behave atypically when compared to outbred animals.

Immunisation with OUR T88/3 protected six out of seven outbred pigs after challenge with OUR T88/1, whereas all of the Babraham pigs exhibited clinical signs, and seven out of twelve reached their humane endpoint. Principle differences between the two experiments were that the Babraham pigs were challenged three days earlier than the outbred animals and the Babraham pigs were 15 weeks old when immunised, whereas the outbred animals were 8 weeks old. The timing of the challenge is unlikely to explain the differences, as immunisation with genotype I strain 1455 [42] or Pretoriuskop/96/4Δ9GL [26] showed protection against parental challenge from 14 days post-immunisation. In the limited number of studies available, older age tends to correlate with better outcomes following ASFV infection [43,44,45,46], and although similar experiments with vaccinated animals have not been performed, it seems age is also unlikely to explain the differences we observed. Magnitude and kinetics of viraemia and clinical signs after challenge with OUR T88/1 of the control group of Babraham pigs immunised with PBS were comparable to previous data obtained in outbred pigs immunised with replication-deficient adenovirus expressing GFP [33,47]. The pigs immunised with the adenoviruses were of a similar age to the Babraham pigs when challenged, and therefore, there is no suggestion that Babraham pigs were more susceptible or sensitive to ASFV. The outbred and Babraham pigs were not bred on the same farm, and therefore, it is possible that environmental factors could play a role in the differences in the performance of OUR T88/3, as has been seen previously with pigs of different health statuses [48,49,50,51]. However, viral shedding and immune responses after influenza A virus infection in Babraham pigs were indistinguishable from those in outbred pigs [32] and health status plays a role in influenza progression in pigs [52,53].

Therefore, taken together, it seems likely that the difference in clinical outcome after challenge with OUR T88/1 was due to the quality of the immune response induced in the Babraham line compared to the outbred animals. Therefore, host genetics may play a role in the differences observed, and the Babraham pigs and NIH *cc* minipigs [18] represent two inbred models that behave atypically after immunisation with the low virulent OUR T88/3 isolate. ASFV-specific antibody and T-cell responses were generated in both Babraham and outbred pigs after immunisation with OUR T88/3. However, both ASFV-specific antibody titres and IFNγ secreting cells were lower in the Babraham pigs than in outbred animals prior to challenge with OUR T88/1. As influenza-specific immune responses in Babraham pigs are broadly comparable to those seen in outbred animals [32], it would be interesting to see if ASF-specific immune responses induced by other attenuated strains of ASFV or pools of adenovirus [33,47] produced similar results in Babraham pigs to those seen in outbred animals.

Numbers of circulating lymphocyte populations in both Babraham and outbred pigs prior to challenge were broadly similar to analogous cell types characterised in a detailed study of naïve outbred Landrace × Large White × Pietrain pigs [54]. Some differences of note were observed though; Talker et al. reported ~3 × 10^6^ CD3+CD4+ cells between 5 and 25 weeks of age, whereas we saw approximately 1.2 × 10^6^ CD3+CD4+ cells in the outbred animals and less than 1 × 10^6^ in the Babraham pigs. Likewise, we report approximately 2 × 10^5^ CD8α+CD4- cells in the Babraham pigs, whereas Talker et al. reported 1.5 × 10^6^ CD3+CD8α+CD8β+ cells/mL, and by gating on CD8α^hi^ cells, we identified approximately 1 × 10^6^ cells/mL in the outbred animals (not shown).

Most classes of mononuclear cells diminished in the number of pigs suffering clinical signs of ASF and then recovered along with the animals. This was consistent with previous reports of lymphopenia in pigs infected with a number of different ASF isolates [44,55,56,57]. No decreases in the number of circulating lymphocytes were observed after immunisation with OUR T88/3, which contrasts with a temporary decrease in pigs infected with the attenuated E75-CV1 and moderately virulent Estonia 2014 strain [56,58]. Infection of domestic pigs with the genotype I non-haemabsorbing NHP/68, which is 99.99% identical to OUR T88/3 [59], induced enhanced levels of NK activity in animals that were ultimately protected from virulent challenge [11]. Although we did not see an increase in the absolute number of circulating CD3-CD8α+ NK cells in either Babraham or outbred pigs, we did not include markers such as CD16 that would enable us to precisely identify NK cells or their activation status [60] and, therefore, cannot rule out a role in the clinical outcomes in our studies. Recent data have shown that numbers of iNKT cells increase after infection with virulent ASFV [61], and it would be interesting to examine both NK and NKT cells in the context of immunisation and challenge.

Babraham pigs immunised with OUR T88/3 exhibited increased numbers of CD4+CD8α+ compared to the control group, and this became particularly apparent in the first few days after challenge. Numbers of CD4+CD8α+ cells then began to drop as clinical signs became evident. This was in contrast to previous data with the virulent Armenia strain of the virus, where elevated numbers of CD4+CD8α cells were observed five days post-infection [57]. We did not see elevated numbers of these cells in either control Babraham pigs challenged with OUR T88/1 or in the naïve outbred pigs challenged with Georgia 2007/1. Although the Armenia strain of ASFV is practically identical to the Georgia 2007/1 strain, different challenge models were used with Hühr et al. using oronasal infection, which would more closely mirror natural pig-to-pig transmission as opposed to the intramuscular challenge reported here. The experiments with Armenia showed clear lymphopenia, and therefore, it is interesting that a different challenge model showed a markedly different effect on this particular cell type.

CD8α+CD4- and CD4+CD8α+ T-cells were the predominant subclass of cells secreting IFNγ after stimulation with challenge virus. Elevated numbers of both cell types were observed in both Babraham pigs and outbreeds recovering from OUR T88/1; interestingly, high numbers of either cell type were not seen in the three outbred animals that recovered from Georgia. Numbers of ASFV-specific IFNγ secreting cells measured by ELISpot or ICS did not predict protection against OUR T88/1 in the Babraham pigs or outbred pigs, as AV74 and AV75 both showed clinical signs and had comparable responses to AV76 and AV79 that did not. However, there were clear differences in the cellular responses of outbred pigs that survived Georgia compared to those that did not, and these were most clearly observed in the response from CD8α+CD4- cells. Notably, the secretion of cytokines by CD8α+CD4- cells after stimulation with OUR T88/1 twenty-one days post immunisation predicted protection against Georgia 2007/1 as well as the pre-Georgia ICS and proliferation assays. Our results are consistent with the results of Monteagudo et al. in that proliferation of CD8α+CD4- cells provided the best prediction of inter-genotype I cross-protection [12]. Attempts to further classify ASFV-specific T-cells prior to Georgia challenge may have been complicated by the observation that two animals (AV74 and AV75) were still viraemic although numbers of circulating CD4+CD8α+ and CD8α+CD4- T-cells had returned to baseline levels. Nonetheless, phenotyping with CD25, CD62L and CCR7 did not reveal any clear pattern relating to the presence of circulating virus or the subsequent clinical outcome after Georgia challenge. Similar patterns of CD25 and CD62L expression on classical swine fever virus-specific CD8α+CD4- T-cells were observed after immunisation with the C-strain live attenuated classical swine fever vaccine [62]. Similarly, ASFV-specific CD4+CD8α+ were predominately CD62L and CCR7- and are likely consistent with CD4+CD8α+CD27- effector memory cells [63].

Considering the original intent was to test the validity of the Babraham line as a model to study ASFV-specific cellular immunity, it was interesting that differences in the antibody response more closely matched the differences in clinical outcome after challenge. Babraham pigs that did not recover had lower antibody titres than those that did. Outbred animal AV77 had an antibody titre similar to those of the Babraham pigs that did not recover, although AV77 also had a poor cellular response as well. All of the remaining outbred animals that were protected against OUR T88/1 challenge had higher ASFV-specific antibody titres than the Babraham pigs that were not protected. Taken together, this suggested a correlation, albeit a weak one, for a role of antibodies in protection against a homologous virulent virus. A number of ASFV proteins have been identified as targets for the antibody response [64,65,66], and results from the competitive ELISA against CP204L/p30 suggested a potential difference in the antibody response to this protein between the animals that recovered and those that did not (Figure 5A). A deeper analysis of the diversity and magnitude of the antibody repertoire to ASFV may reveal patterns that could help infer protection and guide vaccine design. Such analysis will also benefit from the development of robust assays to study antibody function.

## Data Availability

The data presented in this study are openly available in Zenodo at https://doi.org/10.5281/zenodo.6794942, accessed on 9 June 2022.

## Supplementary Materials

The following supporting information can be downloaded at: https://www.mdpi.com/article/10.3390/v14071487/s1, Figure S1: Whole blood T-cell gating, Figure S2: Whole blood B-cell gating, Figure S3: Gating strategy for Babraham ICS, Figure S4: Gating strategy for outbred ICS, Figure S5: Gating strategy for outbred day 42 T-cell phenotyping, Figure S6: Gating strategy for outbred day 42 proliferation, Figure S7: Clinical and virological parameters of individual Babraham pigs, Figure S8: Gamma-delta blood cell numbers in Babraham pigs, Figure S9: Serum levels of cytokines in Babraham pigs, Figure S10: Georgia responsive T-cell phenotypes pre-challenge; Table S1: Whole blood T-cell panel, Table S2: Whole blood B-cell panel, Table S3: Babraham ICS panel, Table S4: Outbred ICS panel, Table S5: Outbred T-cell phenotyping panel, Table S6: T-cell proliferation panel.
