# Supplementary material for: Cellular and Humoral Immune Responses after Immunisation with Low Virulent African Swine Fever Virus in the Large White Inbred Babraham Line and Outbred Domestic Pigs
Source: Viruses. 2022 Jul 7;14(7):1487. doi: 10.3390/v14071487 (PMC9322176; doi:10.3390/v14071487)
Supplement: Supplementary file 1 [file viruses-14-01487-s001.zip › viruses-1788768-supplementary.pdf]

Table S1: Whole blood T-cell panel

Antigen	Clone	Conjugate	Company	Dilution†
CD3	PPT3	Biotin (Strep-BV421)	2B Scientific	1:100 (1:500)
CD4	74-12-4	PerCPCy5.5	BD	1:40
CD25	K231.3B2	Zenon-AF647	Bio-Rad	1:20*
CD8α	MIL2	FITC	Bio-Rad	1:20
γδTCR	PPT16	Zenon-PE	In house	1:4*
Zombie		NIR	Biolegend	1:100

* Dilution of Zenon conjugate, where 5 µl of primary antibody was mixed with 1 µl of each Zenon followed by 1 µl of stop.

† Final dilution when mixed with blood, that in parentheses indicates dilution of streptavidin conjugate.

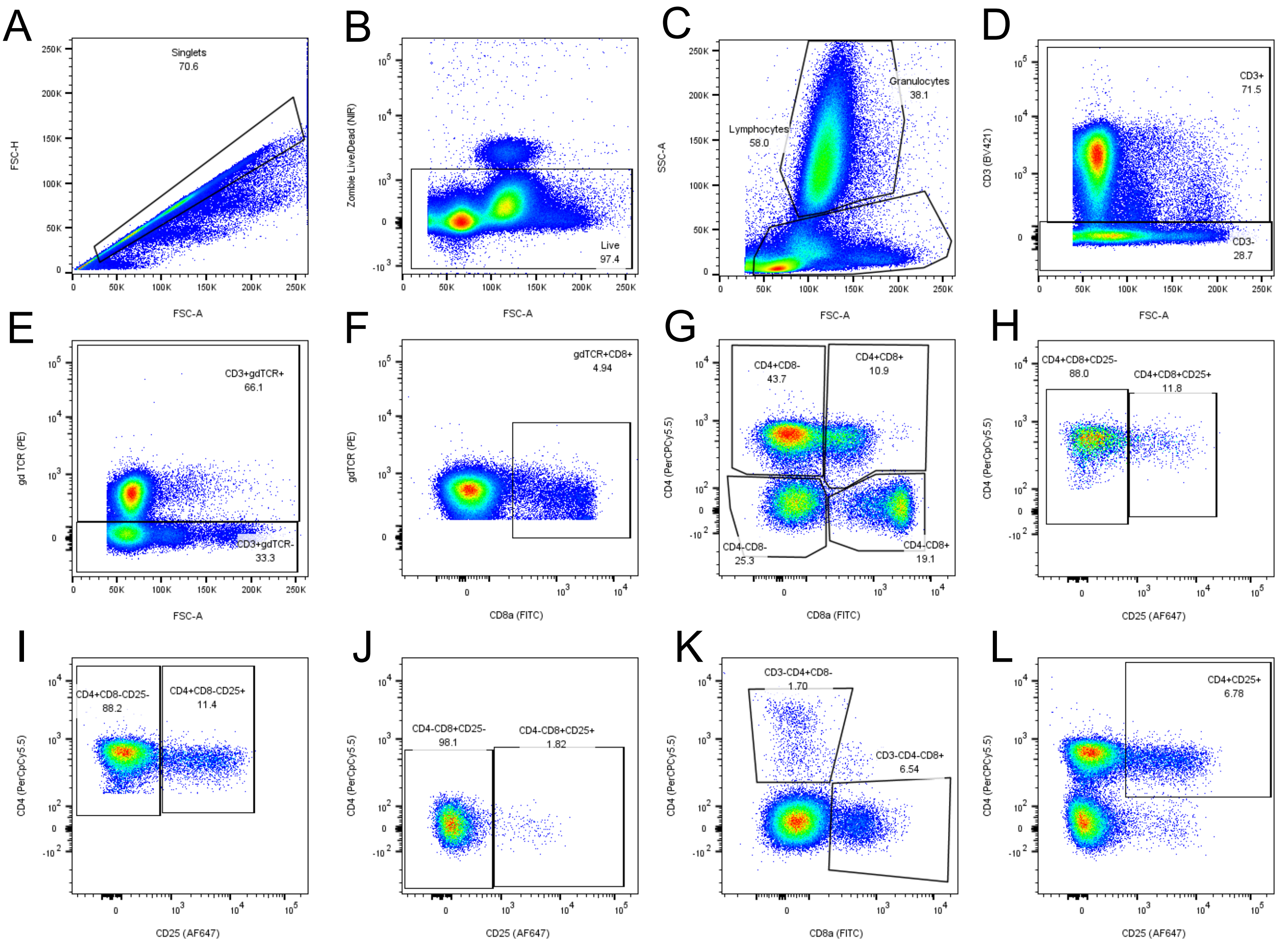

Figure S1: Whole blood T-cell gating. Cell subsets were identified in whole blood samples following the exclusion of doublets (A), the exclusion of dead cells (B), and the selection of lymphocytes (C). CD3⁺ (D) were separated by γδTCR (E) and the γδTCR- population into four populations based on the expression of CD4 and CD8α (F). Activated γδTCR+ cells were identified by the expression of CD8α (F). CD3⁺ cells were divided into CD4⁺, CD8⁺, and CD4⁺CD8⁺ (G), as well as γδ TCR ⁺ (H) and CD25+ cells were identified within the CD4+CD8α+, CD8α+CD4- and CD4-CD8α- populations (G to I). All samples were run on the MACSQuant 10 and analysed using FlowJo v10.6. Representative gating from Pig 917 three days post immunisation.

Table S2: Whole blood B-cell panel

Antigen	Clone	Conjugate	Company	Dilution†
CD21	BB6-11C9.6	Biotin (Strep-BV421)	Novus (Bio-Techne)	1:160 (1:500)
Zombie		NIR	Biolegend	1:100

† Final dilution when mixed with blood, that in parentheses indicates dilution of streptavidin conjugate.

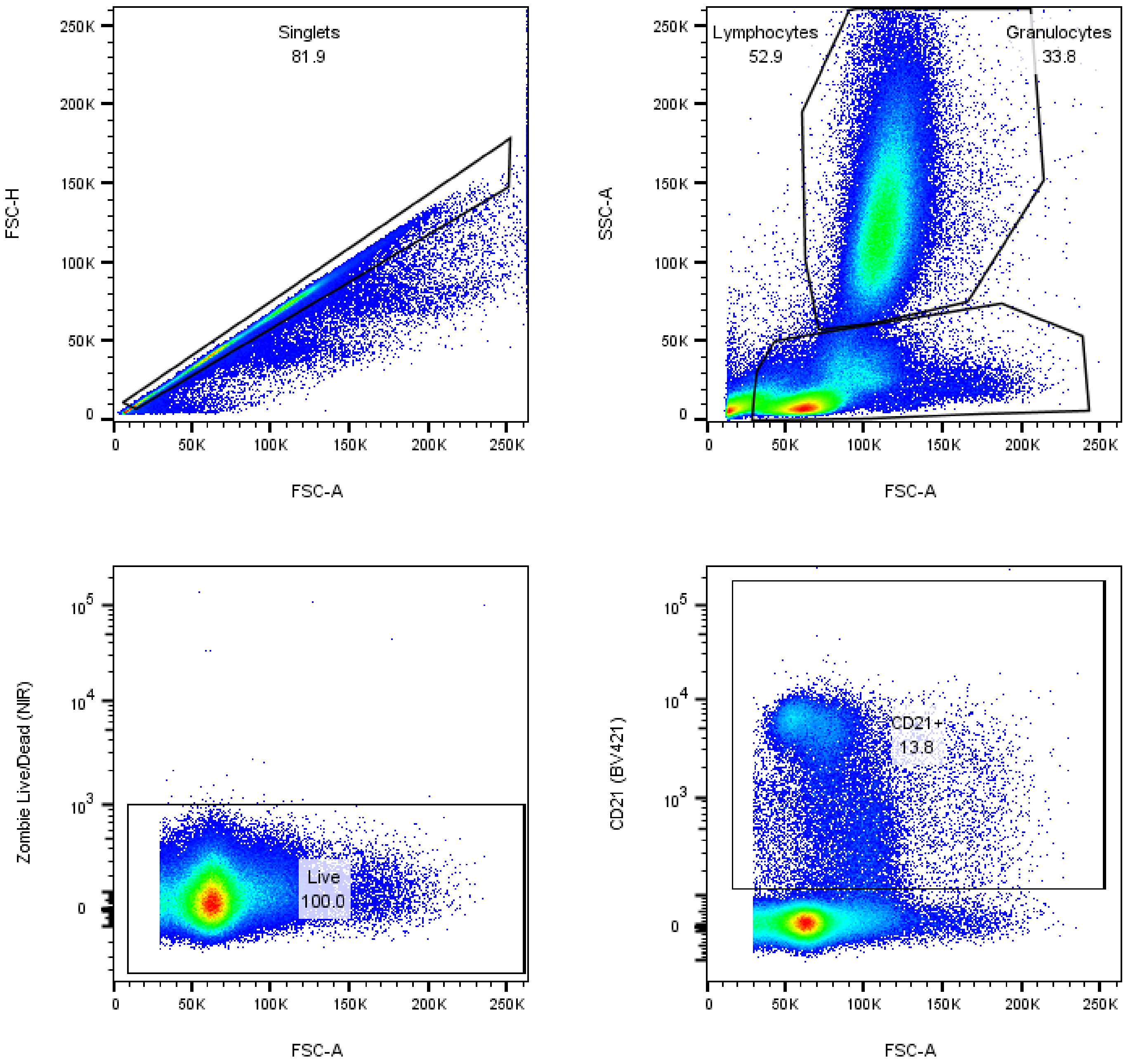

Figure S2. Whole blood B-cell gating B cells were identified in whole blood samples following the exclusion of doublets (A), selection of lymphocytes (B) and exclusion of dead cells (C). B cells were defined as CD21⁺(D). All samples were run on the MACSQuant 10 and analysed with FlowJo v10.6. Representative gating from Pig 917 three days post immunisation.

Table S3: Babraham ICS panel

Antigen	Clone	Conjugate	Company	Dilution
CD3	BB23-8E6-8C8	PE-Cy7	BD	1:20
CD4	74-12-4	PerCPCy5.5	BD	1:20
CD8α	MIL2	FITC	Bio-Rad	1:40
CD8β	PPT23	PE	Bio-Rad	1:5
γδTCR	PPT16	Zenon-AF647	In house	1:4*
IFNγ	P2C11	Biotin (Strep-BV650)	Thermo	1:500 (1:500)
TNFα	Mab11	BV421	Biolegend	1:20
Zombie		NIR	Biolegend	1:100

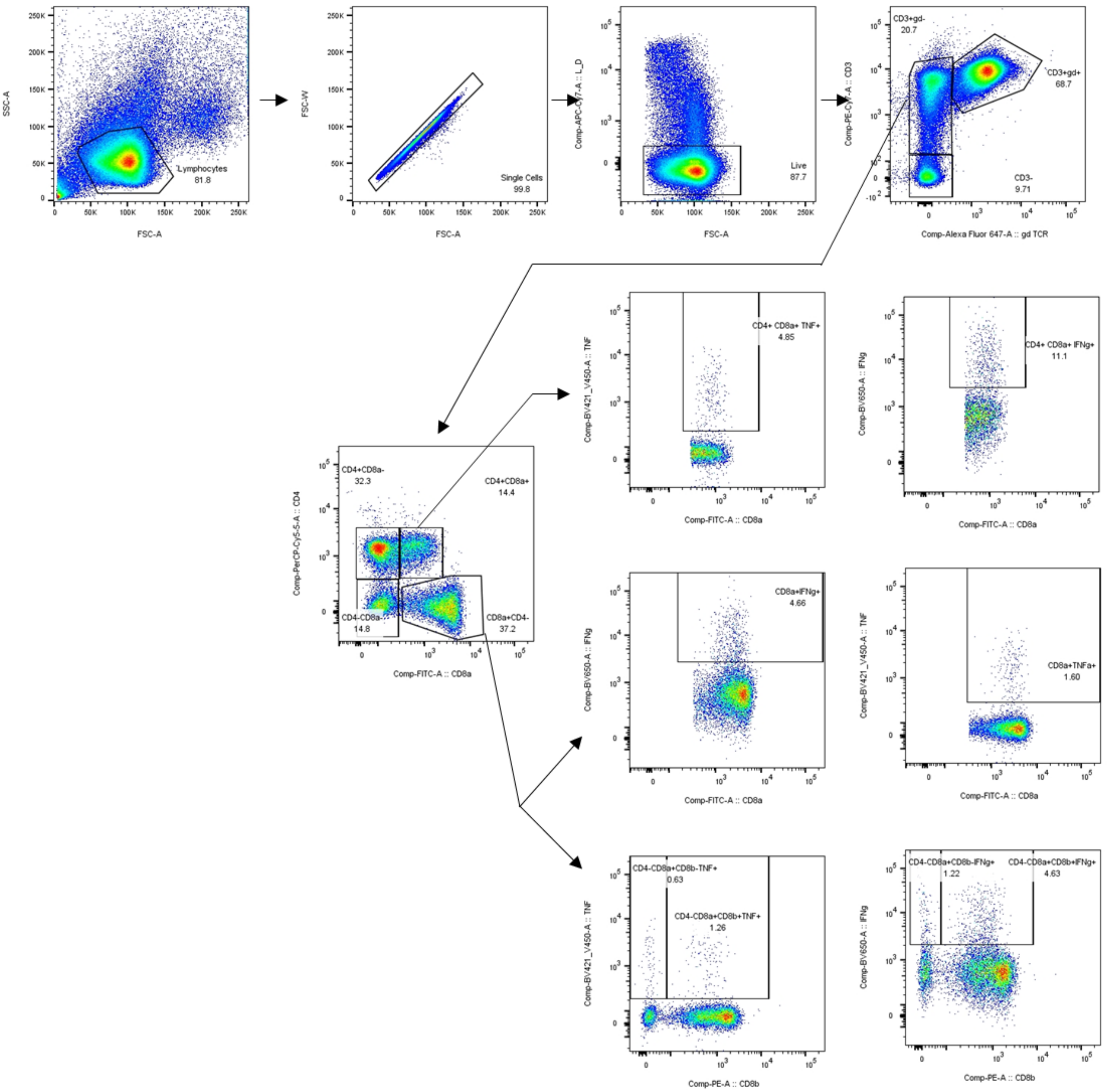

Figure S3: Gating strategy for Babraham ICS. Single live cells were gated on CD3 and γδTCR. CD3+ γδTCR- cells were then further subdivided by CD4 and CD8α, and CD8α+CD4- cells were then gated on the presence of CD8β. Within the populations of CD4+CD8α, CD8α+CD4- and CD8α+CD4-CD8β cells the percentage positive for TNFα and IFNγ were determined. Cells from AV72 stimulated with OUR T88/1 are shown as an example.

Table S4: Outbred ICS panel

Antigen	Clone	Conjugate	Company	Dilution
CD3	BB23-8E6-8C8	PE-Cy7	BD	1:20
CD4	74-12-4	PerCPCy5.5	BD	1:20
CD8α	MIL2	FITC	Bio-Rad	1:40
Perforin	δ7	PECF594	BD	1:5
γδTCR	PPT16	Zenon-AF647	In house	1:4*
IFNγ	P2C11	Biotin (Strep-BV650)	Thermo	1:500 (1:500)
TNFα	Mab11	BV421	Biolegend	1:20
Zombie		NIR	Biolegend	1:100

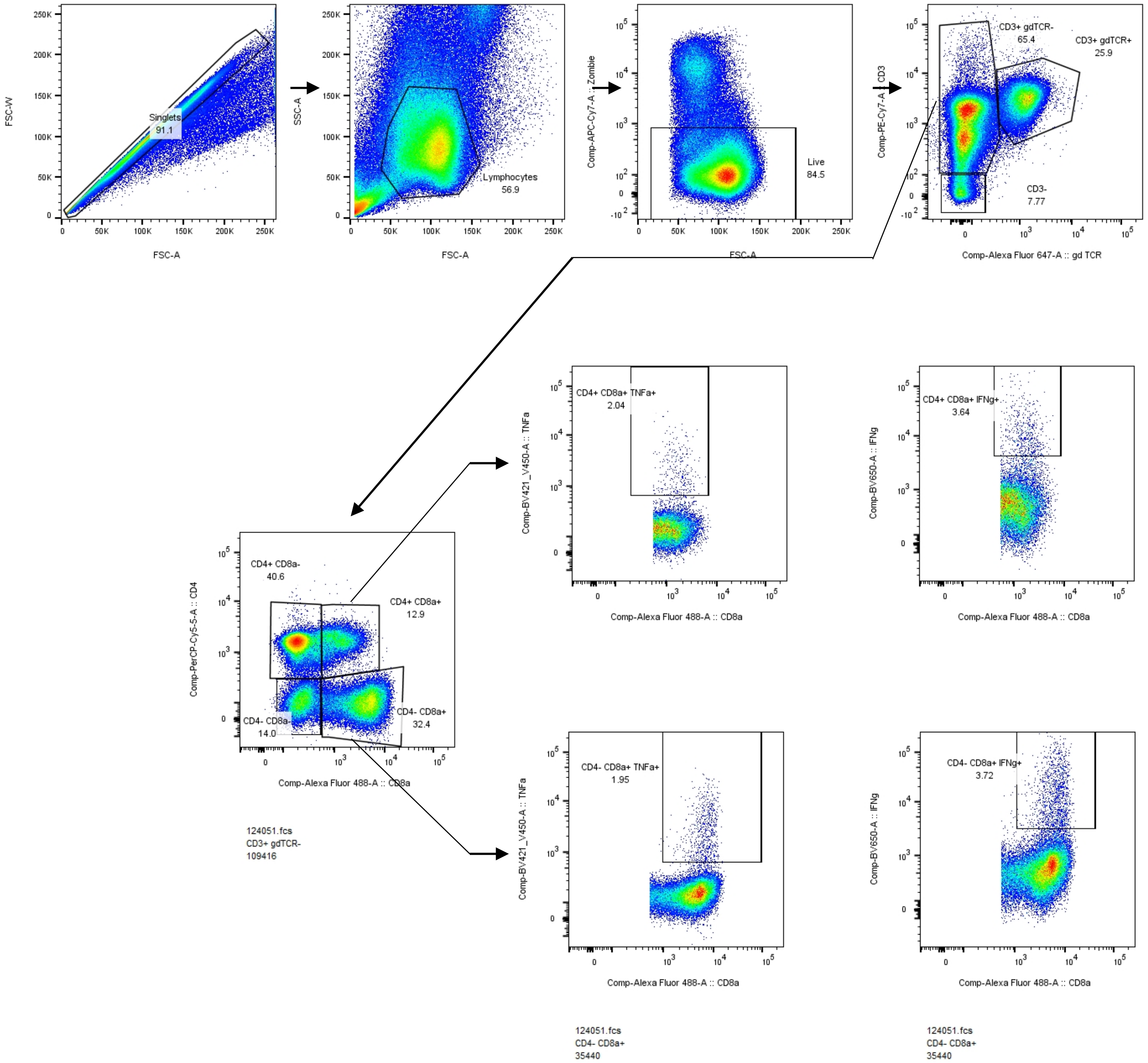

Figure S4: Gating strategy for outbred ICS. Single live cells were gated on CD3 and γδTCR. CD3+ γδTCR- cells were then further subdivided by CD4 and CD8α. Within the populations of CD4+CD8α and CD8α+CD4- cells the percentage positive for TNFα and IFNγ were determined. Cells from AV72 stimulated with OUR T88/1 are shown as an example.

Table S5: Outbred T-cell phenotyping panel

Antigen	Clone	Conjugate	Company	Dilution
CD3	BB23-8E6-8C8	PE-Cy7	BD	1:20
CD4	74-12-4	PerCPCy5.5	BD	1:20
CD8α	295/33-25	PE	BD	1:100
CD25	K231.3B2	Zenon-AF647	Bio-Rad	1:20*
CD62L	CC32	FITC	Bio-Rad	1:12.5
IFNγ	P2C11	Biotin (Strep-BV650)	Thermo	1:500 (1:500)
TNFα	Mab11	BV421	Biolegend	1:20
CCR7	4B12	BV711	Biolegend	1:20
Zombie		NIR	Biolegend	1:100

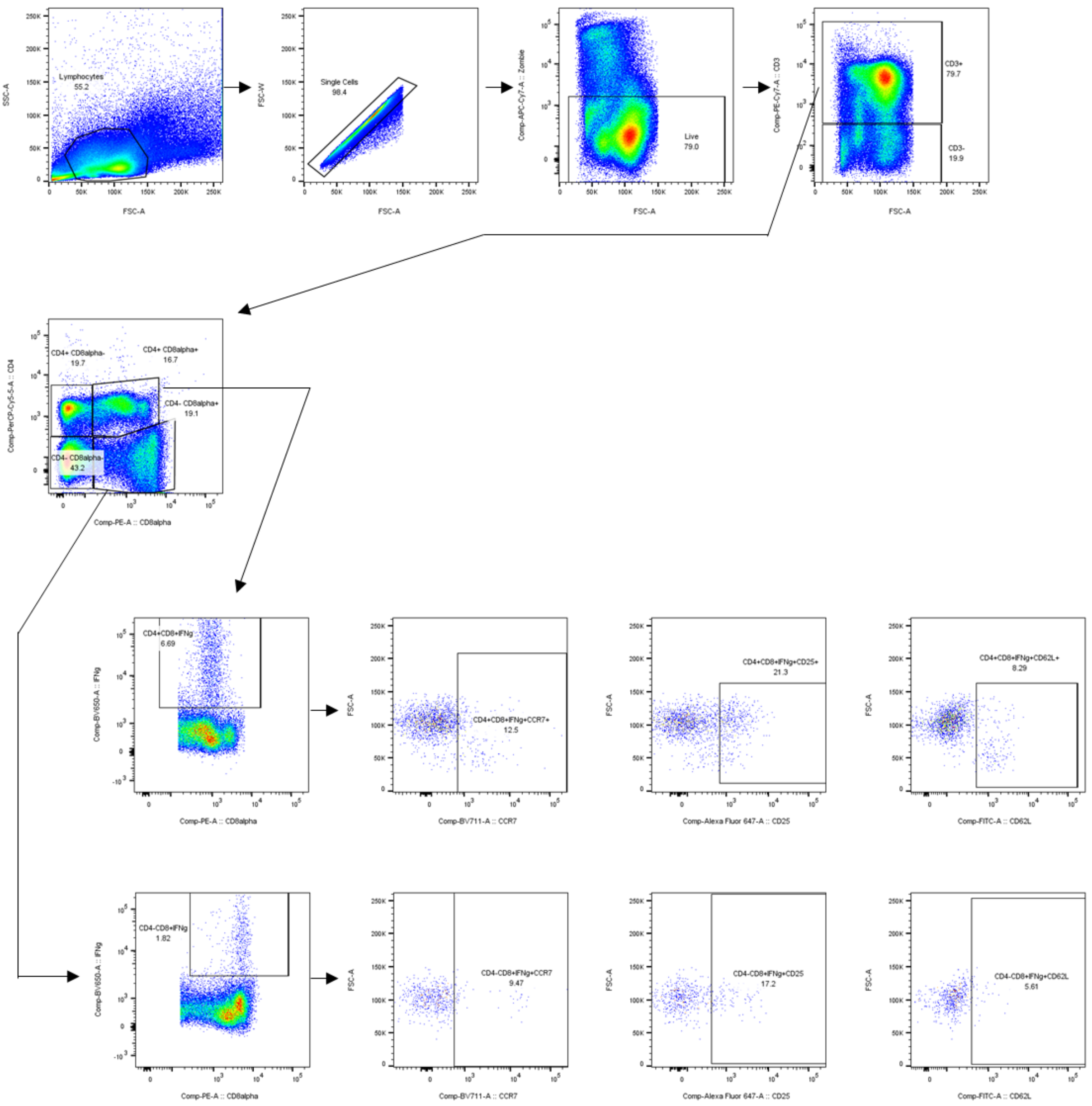

Figure S5: Gating strategy for outbred day 42 T-cell phenotyping. Single live CD3 cells were then further subdivided by CD4 and CD8α and ASFV specific cells defined by the expression of IFNγ. Within the populations of IFNγ+, CD4+CD8α and CD8α+CD4- cells the percentage of CCR7, CD25 and C62L were determined. Cells from AV72 stimulated with Georgia 2007/1 are shown as an example.

Table S6: T-cell proliferation panel

Antigen	Clone	Conjugate	Company	Dilution*
CD3	BB23-8E6-8C8	PE-Cy7	BD	1:20
CD4	74-12-4	PerCPCy5.5	BD	1:20
CD8α	MIL2	FITC	Bio-Rad	1:40
γδTCR	PPT16	Zenon-AF647	In house	1:4*
Zombie		NIR	Biolegend	1:100

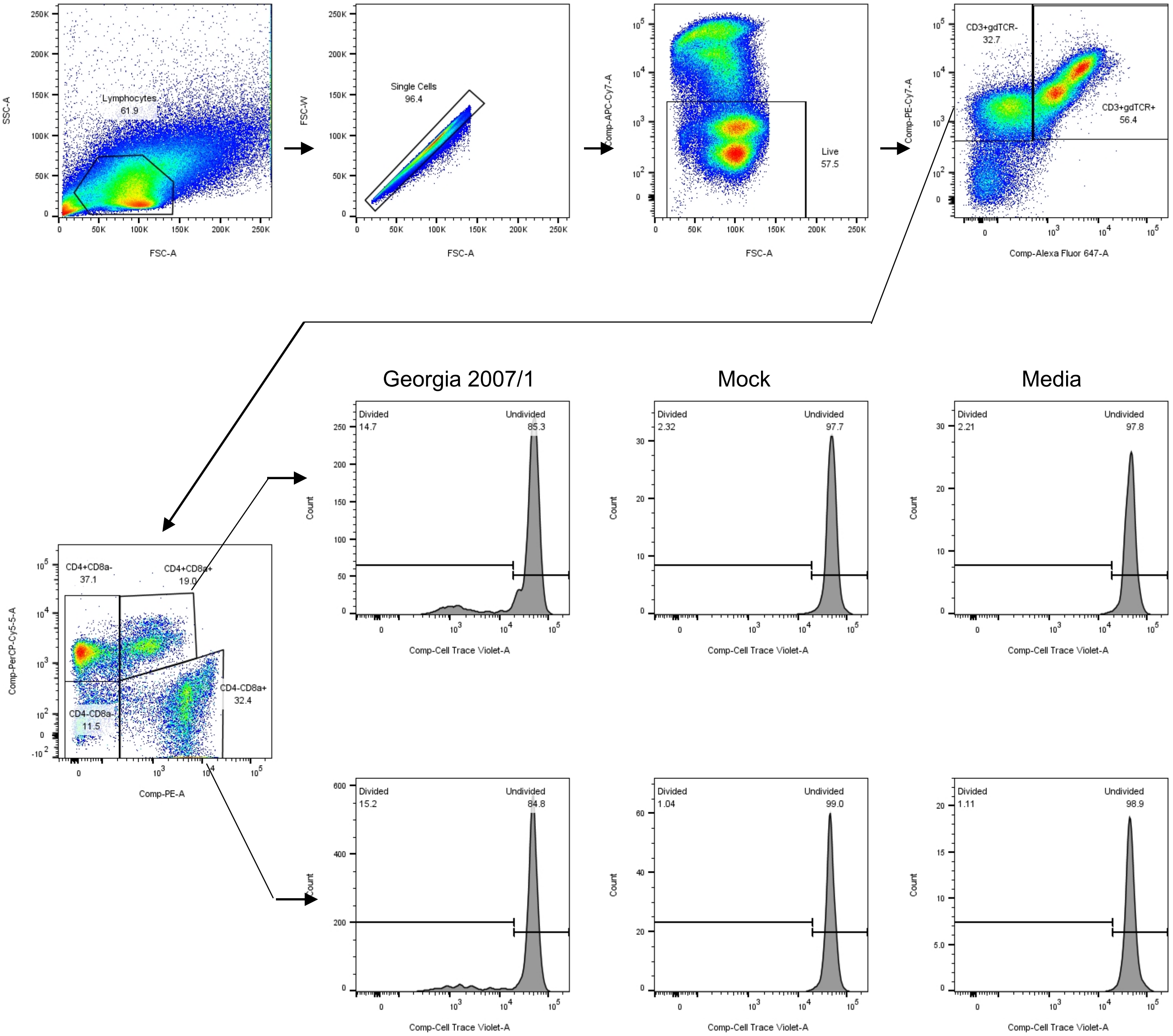

Figure S6: Gating strategy for outbred day 42 proliferation. Single live cells were gated on CD3 and γδTCR. CD3+ γδTCR- cells were then further subdivided by CD4 and CD8α. Within the populations of CD4+CD8α and CD8α+CD4- cells the percentage of divided cells were determined. Cells from AV72 stimulated with Georgia 2007/1 are shown in the dot plots as an example. Histograms show cells from AV72 stimulated with Georgia 2007/1, Mock and Media.

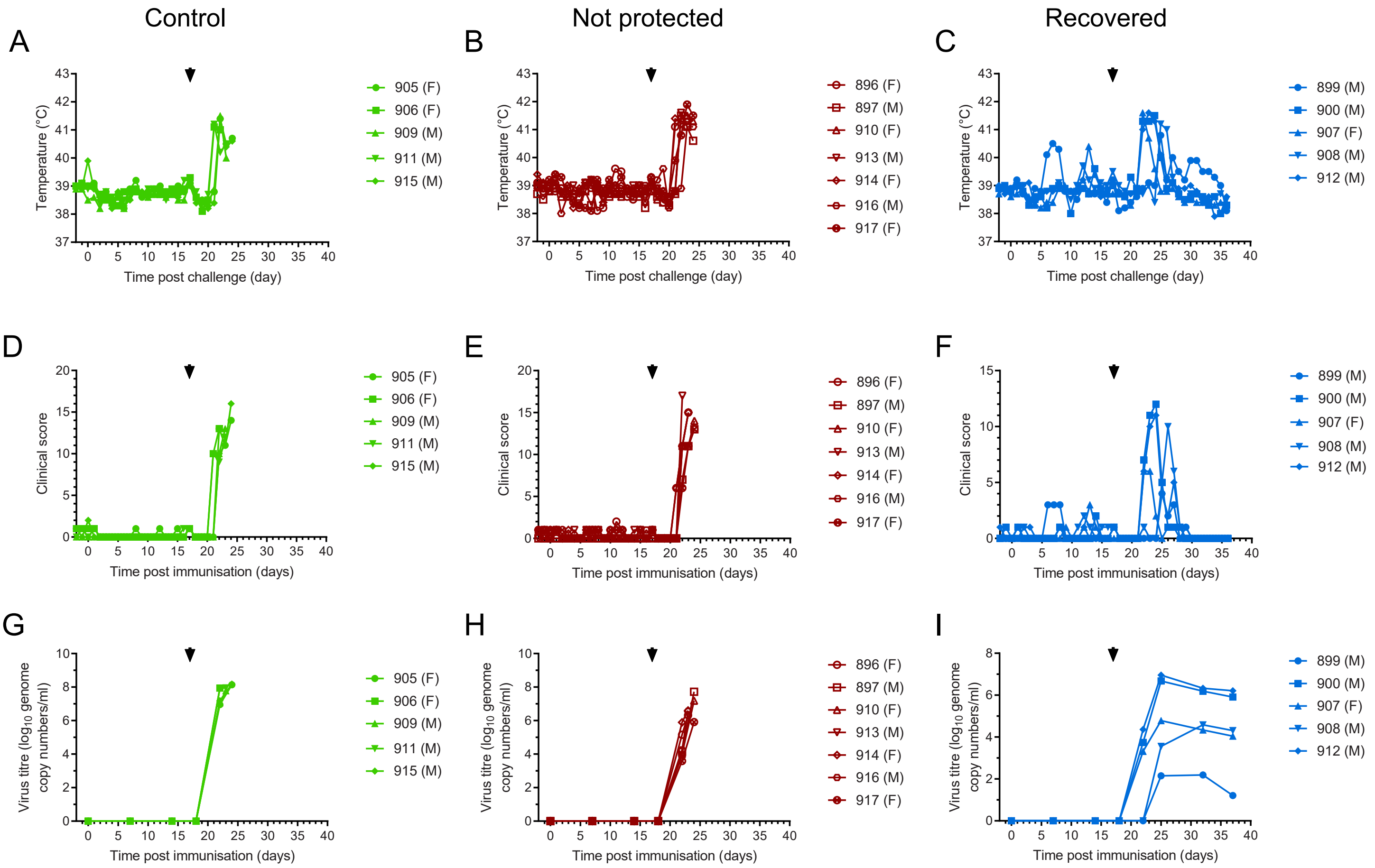

Figure S7: Clinical and virological parameters of individual Babraham pigs. Inbred Babraham animals were inoculated with low virulent ASFV isolate OUR T88/3 (red and blue) or PBS control (green) and then challenged with highly virulent OUR T88/1 eighteen days later (arrows). Body temperatures (A-C) and clinical signs (D-F) were scored daily and blood samples taken for virus titration on the indicated days (G-I). Viremia was determined by quantitative PCR and the sex of each animal is indicated.

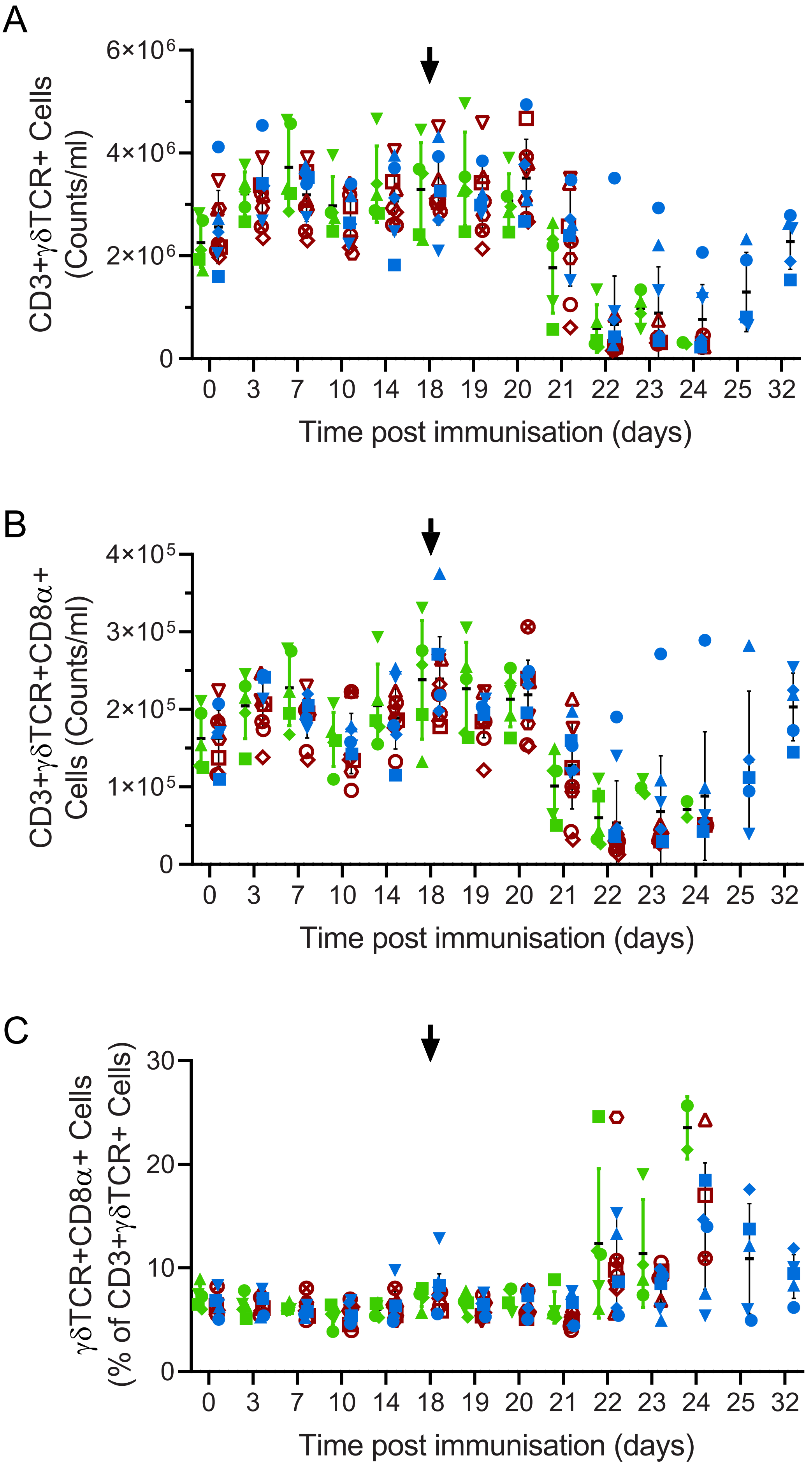

Figure S8: Gamma-delta blood cell numbers in Babraham pigs. Inbred Babraham animals were inoculated with low virulent ASFV isolate OUR T88/3 (red and blue) or PBS control (green) and then challenged with highly virulent OUR T88/1 eighteen days later (arrows). Blood samples were taken on the indicated day, labelled with antibodies and the number of CD3+γδTCR+ (A) and CD3+γδTCR+CD8α+ T-cells (B) determined by volumetric flow cytometry. The proportion of γδTCR+ T-cells expressing CD8α was also determined (C). Animals immunised with OUR T88/3 that recovered after challenge are indicated as blue symbols and those that did not as dark red symbols. Lines indicate the mean and error bars the standard deviation from that mean.

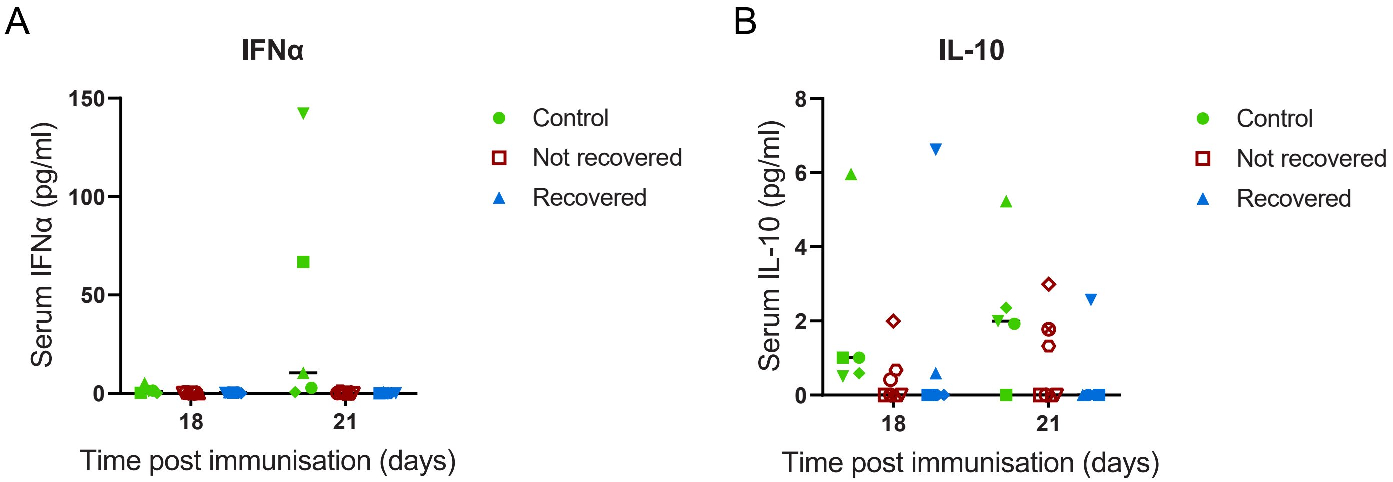

Figure S9. Serum levels of cytokines in Babraham pigs. Inbred Babraham animals were immunised with low virulent ASFV isolate OUR T88/3 (red and blue) or PBS control (green) and then challenged with highly virulent OUR T88/1 eighteen days later. Blood samples were taken prior to challenge (Day 18) or three days later (Day 21) and levels of IFNα (A) and IL-10 (B) determined by ELISA. Lines indicate the mean.

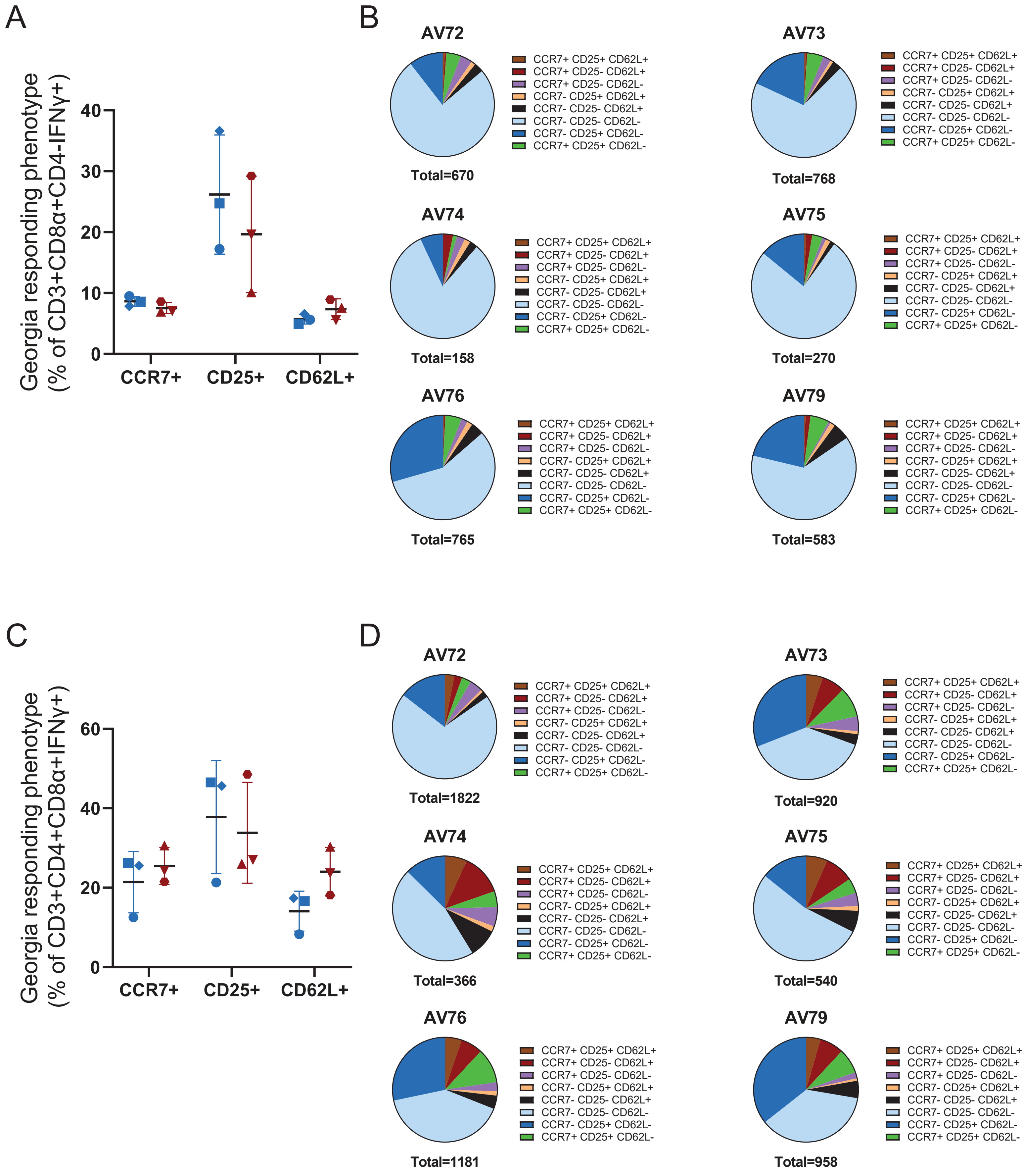

Figure S10: Georgia responsive T-cell phenotypes pre-challenge. Pigs were immunised with OUR T88/3, boosted with OUR T88/1 three weeks later and PBMCs collected three weeks after boost (Day 42). Cells were stimulated overnight with Georgia 2007/1 and then labelled with CD3, CD4, CD8α, IFNγ, and activation markers CD25, CD62L and CCR7. Panel A and C show the proportion of IFNγ+ cells expressing CD25, CD62L and CCR7 on CD8α+CD4- and CD4+CD8α+ cells respectively and Panel B and D show the proportions of the different combinations of CD25, CD62L and CCR7 on the two cells types.
